# Case Report: First Documented Hip Arthroplasty on Chinese Patient with Ochronotic Arthropathy

**DOI:** 10.3389/fsurg.2022.875777

**Published:** 2022-05-09

**Authors:** Zhibo Ying, Jufeng Lu, Xucheng Wang, Qinghe Zeng, Hongting Jin, Bangjian He

**Affiliations:** ^1^ Zhejiang Chinese Medical University, Hangzhou, Zhejiang Province, China; ^2^ Zhuji Sixth People’s Hospital, Shaoxing City, China; ^3^ First Affiliated Hospital, Zhejiang Chinese Medical University, Hangzhou, China

**Keywords:** alkaptonuria, ochronotic arthropathy, total hip arthroplasty, autosomal recessive disorder, homogentisc acid

## Abstract

Alkaptonuria (AKU) is a rare autosomal recessive disorder caused by homogentisc acid (HGA) accumulation, the deposition of which in the joints usually causes ochronotic arthropathy. With no specific therapy for AKU currently, total joint arthroplasty in ochronotic arthropathy is applied to relieve the symptoms. A 63-year-old female patient came to our Orthopedic Surgery Department in 2019, complaining of severe limitation of movement and pain in the right hip for more than one year. A right total hip arthroplasy (THA) was performed due to the ineffective conservative therapy. At a follow-up of more than 15 months, the woman had full mobility with no complaining of pains. Since there is no relevant case reported about THA therapy for Chinese AKU patients, this report provides a feasible scheme, which makes clinical data more comprehensive.

## Background

Alkaptonuria (AKU) is a rare, inborn, metabolic disease characterized by accumulation of homogentisc acid (HGA) affecting only 1 in 200,000 to 1,000,000 individuals worldwide, which was first depicted by Garrod in 1908 as 1 of the 4 inborn errors of metabolism (1). Deposition of oxidized HGA in connective tissue generates dark discoloration joints known as black joints disorder (ochronosis or ochronotic arthropathy). Accumulated HGA is then excreted in urine and the urine is transformed into dark black or brown on alkalinization and oxygenation. Patients usually have no obvious symptoms in childhood. Nevertheless, ochronosis may begin to appear as a brown or dark pigmentation around the sclera or pinna; renal or prostatic stones; lower back or joints pain; osteoporosis; fractures; or rupture of the tendons in the 40–60 years of life (2).

This disorder usually affects the knee and hip joints, which subsequently causes severely degenerative arthritis, significantly impeding patients’ normal life.

Most of the cases reported in China were about knee joints, which illustrated that total knee arthroplasty (TKA) had satisfactory outcomes (3). Due to the scarcity of hip-affecting cases, whose prognoses are unclear, this case provides a special perspective for the treatment and rehabilitation of hip joints in those with ochronotic arthropathy.

## History and Physical Examination

**Figure 1:**
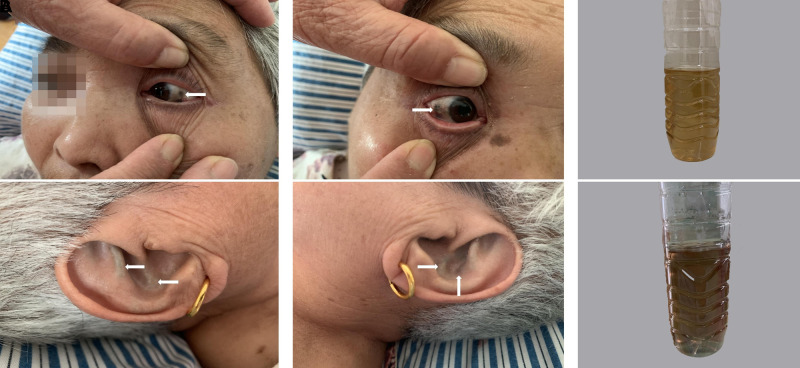
Dark brown pigmentation in physical examination shows characteristic of Ochronosis in bilateral sclera; dark pigmentation observed on bilateral ear auricle (**A**). A urine sample at different times: (**A**) fresh urine; (**B**) fresh urine exposed to air for 4 h. Urine darkening can be observed (**B**).

A 63-year-old female was complaining of severe limitation of movement and pain in the right hip for more than one year. The patient suffered pain particularly during walking and stair climbing and the symptoms were relieved when she took a rest. She underwent left hip arthroplasty 7 years ago in Beijing Jishuitan Hospital due to left femoral head necrosis. Unfortunately, her right hip pain was increasingly severe recently. A more in-depth patient history revealed that she had other diagnoses, including bilateral carotid artery medial tunica intima rough with left side plaque formation, atherosclerosis of aorta and coronary arteries, which made the operation more difficult from other AKU patients. The patient was Chinese, who was married with two children. When asked for her family history, she denied consanguineous marriage. She told us that her urine was black since childhood and her brothers, sisters, and children were all in good health. She had no history of tobacco, alcohol, or addictive drug use. Besides, she had no recent travel or sick contacts. She had no known family history of infectious diseases, inflammatory arthritis or malignancy. Further physical examination revealed that her right hip had no obvious swelling but local tenderness, whose range of motion (ROM) was limited, with 20 degrees of abduction, 15 degrees of adduction, 95 degrees of flexion and 10 degrees of posterior extension. Her Harris Hip Score (HHS) was 56. The right lower limb was about 1.5 cm shorter than the other side, resulting in poor mobility. The subsequent clinical examination revealed dark pigmentation of the sclera of bilateral eyes and on the ear auricle. (Figure 1A) Her urine appeared normal in color (buff), but when exposed to air for about 4 to 6 h, it gradually turned brown (Figure 1B).

## Imaging Evaluation

**Figure 2:**
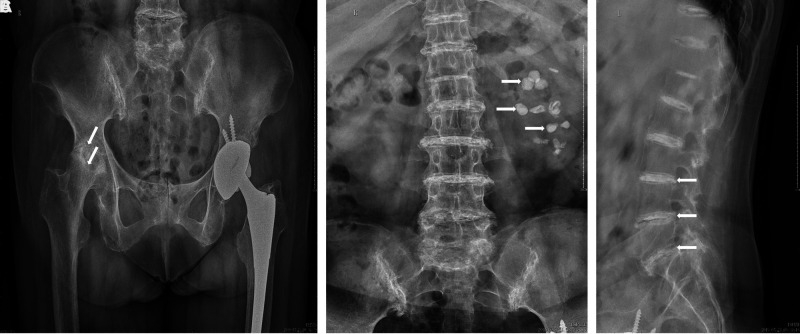
Preoperative X-rays of hip joints showed the density of the right femoral head was increased unevenly with rough joint surface and narrow joint space, the upper acetabulum edge was slightly hyperosteogenic, and the joint was in position after left artificial hip implantation. These features resemble as osteoarthritis (**A**). Some scattered calcifications in the left kidney (**B**) and multilevel narrowing of the intervertebral spaces (**C**).

The former plain radiography results of the hips showed that the acetabular prosthesis shifted to the center, with the joint in good position and alignment, the upper femoral segment osteoporotic, and the stump flat. In addition, the density of the right femoral head was increased unevenly with rough joint surface and narrow joint space, the upper acetabulum edge was slightly hyperosteogenic, and the joint was in position after left artificial hip implantation (Figure 2A). Another slide of the spine illustrated multilevel narrowing of the intervertebral spaces and some scattered calcifications in the left kidney, which contributed to the diagnosis and made ochronotic arthritis distinct from other degenerative joint disease (Figures 2B,C).

## Differential Diagnosis

The above clinical manifestations and imaging findings need to be identified from the following disorders: avascular necrosis of the femoral head, rheumatoid arthritis and ankylosing spondylitis.

## Avascular Necrosis of the Femoral Head

The clinical manifestations of femoral head necrosis are hip pain, such as intermittent pain and walking pain. Preoperative X-ray of the pelvis showed striking avascular necrosis of the right hip, with resorption of the femoral head, and erosion of the superior acetabulum (4). However, the manifestations of our patient were not merely resembled with the features of femoral head necrosis, such as blackened urine, dark pigmentation of the sclera of bilateral eyes and on the ear auricle and dark discoloration hip joints (2). All of these features made it more likely to be diagnosed as ochronotic arthropathy.

## Rheumatoid Arthritis

Rheumatoid arthritis (RA) is a chronic, inflammatory joint disease, which leads to disability, inability to work and increased mortality. The occurrence of RA in women is 2 to 3 times higher than in men (5). Since our patient had chronic hip joint pain, we considered the possible diagnosis of RA. Some of the specific symptoms may indicate possible RA, including autoantibody positivity, joint pain and swelling in metatarsophalangeal joints, metacarpophalangeal joints, or both of them, and morning stiffness of finger joints lasting 30 min or longer (6). This patient, however, did not resemble those specific symptoms, but had other evident symptoms that could be seen in ochronotic arthropathy.

## Ankylosing Spondylitis

Two distinguishing features of ankylosing spondylitis (AS) are the bamboo spine and the formation of extensive fusion between various vertebral bodies of the spine (7). Although almost all patients with AS have different degrees of sacroiliitis, a hallmark of AS (7), complete spinal fusion is rare in clinical practice. Although sacroiliitis, intervertebral space narrowing and vertebral body degeneration were observed in the patient, there was no occurrence of extensive intervertebral fusion and bamboo spine. Besides, two main laboratory indicators are potentially relevant for the diagnosis of spondyloarthritis—HLA B27 and C-reactive protein (8). However, the laboratory tests indicated that both indexes were normal. In summary, the diagnosis of ochronotic arthroplasty was established.

## Interventions and Outcomes

**Figure 3:**
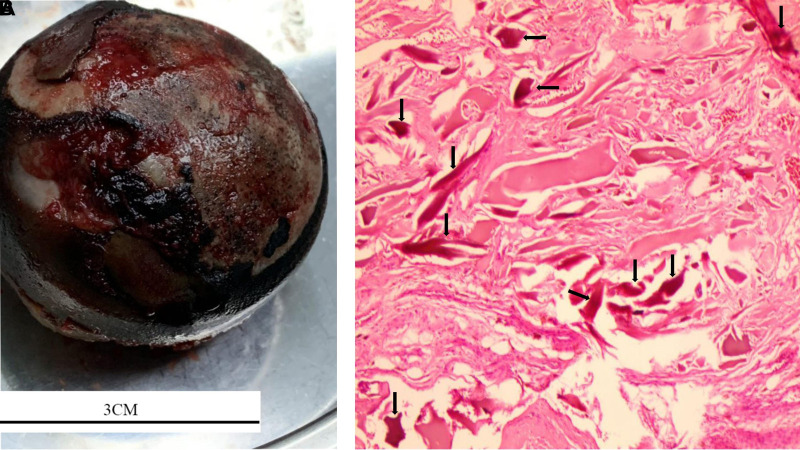
Intraoperative findings showed blackened labrum and femoral head. The articular cartilage was completely stripped off (**A**) Histopathology revealed a large number of histiocytic reactions and multiple pigmented areas. (H&E 10 × 10) (**B**).

**Figure 4:**
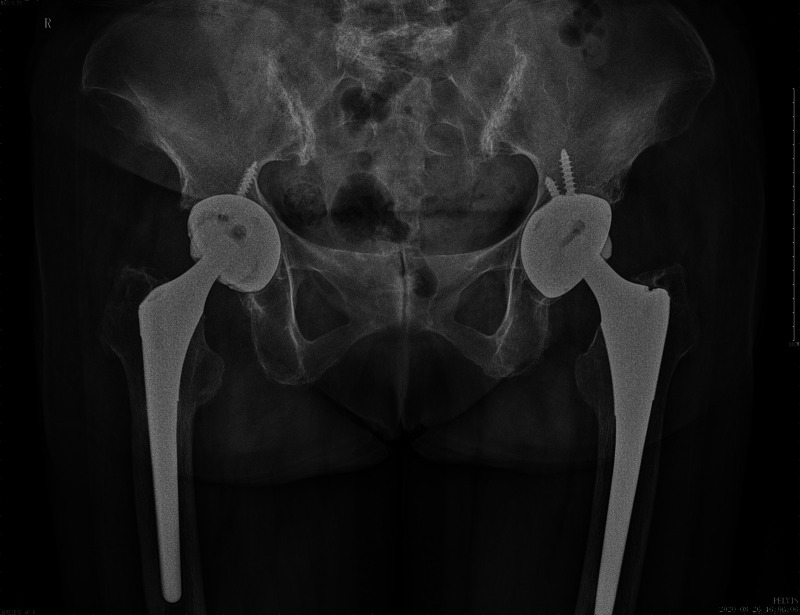
Postoperative X-rays of hip joints showed well integrated and positioned component.

After the patient was admitted, we gave her some nonsteroidal anti-inflammatory drugs (NSAIDS) and did some physical therapy as well, such as ankle pump exercise and hip muscle exercise, those, however, were ineffective. Further physical examination revealed that her Harris Hip Score (HHS) was 56. Since alkaptonuria was a rather systemic disorder, and our patient did have cardiac diseases, we recommended that these patients needed a comprehensive pre-operative assessment. After a series of preoperative examinations, we found that the patient was indeed indicated for surgery. A right total hip arthroplasty (THA) was performed after the informed consent. A poster lateral arc incision about 15 cm in length was made at the right hip joint after disinfection. The skin, subcutaneous and fascial layers were cut in turn, and the gluteus maximus was separated. The synovium capsule of the greater trochanter, the gluteus medius insertion of the greater trochanter and the vastus lateralis insertion were black and silty, with the gluteus maximus insertion partially avulsing. The bursa of the greater trochanter and the blackened tissues were thoroughly cleared with the electric knife, the insertion point of the greater trochanter of the external rotator muscle group was cut off and pulled back and upward to expose the back side of the joint capsule. After the cross incision, the joint capsule was fully exposed, the labrum and femoral head were blackened, and the articular cartilage was completely stripped off (Figure 3A). The femoral neck was removed after vertical cutting 1.2 cm above the minor rotor. Hyperplastic synovial tissue, blackened acetabular pelvis lips, posterior joint capsule and osteophytes were completely removed. The fibrous tissue was released and the acetabulum was exposed. The acetabular cartilage was ground 45 degrees forward to the acetabular true mortar, and extensive bleeding of the acetabular base cartilage was seen. After stopping the bleeding thoroughly and rinsing the acetabulum, the acetabular mold was put in good position and direction. Placing the biotype mortar cup (NO.50) and secured with 2 screws, then the polyethylene lining was placed. The femur side was slotted forward 15 degrees, and bioprosthesis (NO.7) was used after medullary expansion. The range of motion of the hip joint and the prosthesis were stable after mold test was carried out with standard neck (NO. 32). Taking out the test model, and ceramic head of the standard neck (NO. 32) was installed after thorough flushing for hemostasis. After reduction, the mobility and prosthesis were stable again without dislocation. The great trochanter was drilled with a kirschner wire(1.5 mm). The insertion point of external rotator muscle group was reconstructed with Ethibond suture (NO.5). A drainage tube was placed and the incision was sutured layer by layer. Stryker bioprosthesis was used during the procedure. After surgery, Cefuroxime 1.5 g iv drops were used to prevent infection with low molecular weight heparin anticoagulation. Twenty-four hours after the drainage tube was removed, the patient underwent rehabilitation training. Intraoperative tissue samples were sent for histological analysis. Histopathology showed (right femoral head) bone tissue with different sizes of trabeculae, more necrotic bone fragments, interstitial hemorrhage, a large number of histiocytic reactions, fibrous tissue hyperplasia, inflammatory cell infiltration, and hemosiderosis (Figure 3B). The operation was performed without any intraoperative challenges. Postoperative plain radiograph showed total hip components were in good position and alignment (Figure 4).

Functional exercise on the day after operation was carried out step by step after anaesthesia, including active training, which continued ankle pump exercise, quadriceps femoris and gluteus muscle contraction training and passive training, including the guidance to step on the bed, lift the body out of the bed, and the massage of her back, buttocks and lower limbs (from bottom to top). The duration of the above training was 15 min per 2 h.

On the first day to first week after operation, she continued the active training. On the second week after operation, she continued the active training and focused on the muscle strength training of the affected limb. After two weeks in hospital, the patient was discharged, whose rehabilitation training was still engaged after discharge.

Her outcomes after the operation and physical therapies were excellent. At 15 months, she was satisfied with her progress, whose range of motion (ROM) was 40 degrees of abduction, 20 degrees of adduction, 120 degrees of flexion, and 25 degrees of posterior extension. Both of her lower limbs were equal in length, and her right HHS was increased significantly from 56 to 96 after the operation.

## Discussion

Alkaptonuria (AKU) is a rare metabolic disorder, which is caused by homogentisate 1, 2-dioxygenase (HGD) enzymes’ error in an essential amino acid metabolic pathway (1). This enzyme aids in the decomposition of HGA, whose defection leads to HGA accumulation gradually, which then deposits in the tissues and blood, especially the connective tissues. The deposition of those polymers in the connective joints usually causes the dark pigmentation, (9) which is also known as ochronotic arthropathy and is usually asymptomatic until the joint lesion becomes more apparent (10). The main clinical manifestations of our patient were similar to those described in the literature, such as joints pain, dark brown urine when exposed to air for several hours, dark pigmentation around the sclera and pinna and renal stones, which made it possible to tell the difference between ochronotic arthropathy and degenerative osteoarthritis (2). Ochronotic arthritis is characterized by progressive degenerative arthropathy mostly affecting spine and big joints (3). The knee is the most commonly affected joint followed by the hip (10).

For AKU patients, it is advisable to follow a vegetarian diet in childhood and maintain a mild protein restriction with the use of nitisinone during their adulthood, which may slow the progression of this disorder (11). Besides, the spectra data acquired from the non-ochronotic and ochronotic samples are significantly different at the point of data collection, which means that the Raman spectroscopy can be used to sensitively detect ochronosis and monitor its progression (12).

At present, there is no exact treatment, the main treatment is symptomatic treatment. During the early stages of AKU, Raman spectroscopy could be used to monitor its progression. Subsequently, we may use nitisinone as prophylactic treatment. All of these can be used to delay the progression of this disease. However, if the patients were in their advanced stages of AKU, the so-called ochronotic arthropathy stage, the most effective way to control the disease could be joints arthroplasty.

To date, there is no relevant medical record about THA therapy for patient with ochronotic arthropathy in China, and the existed reports are mostly about knee joints, so this case, of great significance, enriches our knowledge about ochronotic arthropathy. Fortunately, we found that THA and phycial therapies had promising outcomes (10). So the conclusion may be drawn that the prognosis of THA therapy for patients with ochronotic arthropathy in China and other countries is similarly satisfactory. Finally, we summarized some shortcomings of this case report: Firstly, this case reported only one patient, so it lacked convincing to a certain extent. Of course, this is because the low incidence of the disease, but we will try to expand the sample size. Secondly, for this patient, the treatment we offered was relatively conventional, but it did play a good therapeutic effect. We hope that in the future intervention can be carried out in the early stages of the disorder and we will be able to treat ochronotic arthropathy with other advanced therapies rather than just surgery. Meanwhile, we will continue to follow up the patient’s recovery.

## Data Availability

The original contributions presented in the study are included in the article/Supplementary Material, further inquiries can be directed to the corresponding author/s.
